# Antibiotic-Induced, Increased Conjugative Transfer Is Common to Diverse Naturally Occurring ESBL Plasmids in *Escherichia coli*

**DOI:** 10.3389/fmicb.2019.02119

**Published:** 2019-09-10

**Authors:** Gang Liu, Karolina Bogaj, Valeria Bortolaia, John Elmerdahl Olsen, Line Elnif Thomsen

**Affiliations:** ^1^Department of Veterinary and Animal Sciences, Faculty of Health and Medical Sciences, University of Copenhagen, Copenhagen, Denmark; ^2^National Food Institute, Technical University of Denmark, Lyngby, Denmark

**Keywords:** antibiotic resistance, extended spectrum beta-lactamases, plasmid transfer, antibiotic induced conjugation, *Escherichia coli*

## Abstract

Previously, we showed that cefotaxime (CTX) exposure increases conjugative transfer of a *bla*_CTX–M–__1_ encoding IncI1 plasmid (IncI1/pST49/CTX-M-1) in *Escherichia coli* in a SOS-independent manner. This study aimed at investigating whether the observation was unique for that plasmid/strain/antibiotic combination or whether antibiotic-induced plasmid transfer (PT) is a more general phenomenon among plasmids in *E. coli*. Whole genome sequences of 25 *E. coli* strains were analyzed to identify different extended spectrum beta-lactamases (ESBL) plasmids enabling selection of a diverse collection of plasmids. Experiments were performed following exposure of these strains to 1/2 minimal inhibitory concentration (MIC) of CTX, ampicillin (AMP), or ciprofloxacin (CIP) before conjugation experiments. The frequency of PT was measured and compared to that of donors not exposed to antibiotics. Reverse-transcribed-quantitative real time polymerase chain reaction (RT-qPCR) was used to measure mRNA levels of five PT genes and two SOS response genes in donors exposed to antibiotics. The PT of eight strains (30.8% of strains tested) with IncI1/pST7/CTX-M-1, IncI1/pST49/CTX-M-1, IncI1/pST3/CTX-M-1, IncI1/pST293/CTX-M-1, IncI1/pST295/CTX-M-1, IncI1/pST16/CTX-M-55, and IncFII/CTX-M-14 (*n* = 2) plasmids was significantly increased following antibiotic exposure. CTX increased PT in all of these eight strain/plasmid combinations, AMP and CIP increased the PT in six and three strains, respectively. RT-qPCR showed that PT genes were up-regulated in the presence of the three antibiotics, whereas SOS-response genes were up-regulated only following CIP exposure. Our findings reveal that antibiotics can increase PT in *E. coli* strains with various ESBL plasmids. Thus, antibiotic-induced conjugative transfer of ESBL plasmids appears to be a common phenomenon in *E. coli*, having important implications for assessing the risks of antibiotic use.

## Introduction

Conjugation is one of the most important mechanisms for intra- and inter-species horizontal gene transfer (HGT), and it plays a significant role to accelerate the dispersal of antibiotic resistance genes (Mazel and Davies, 1999; Barlow, 2009; Halary et al., 2010). Integrative and conjugative elements (ICEs) and conjugative plasmids are both mobile genetic elements that move between cells using conjugation machinery, and the major difference lies in their respective maintenance mechanism within a bacteria cell, as plasmids are extrachromosomal autonomously replicating elements, while ICEs must integrate into chromosomes for stable inheritance (Johnson and Grossman, 2015). Bacterial plasmid conjugation is primarily mediated by type IV secretion systems (T4SSs) and involves direct cell-to-cell contact, mating-pair formation (Mpf), relaxosome formation, and DNA exchange (de la Cruz et al., 2010).

Extended-spectrum beta-lactamase (ESBL) production is a common resistance mechanism against β-lactam antibiotics in Gram-negative bacteria (Al-Bayssari et al., 2015), and the rapid dissemination of novel genes reflects their evolution under the selective pressure of antibiotic usage. ESBL genes are generally acquired by HGT, and they are able to hydrolyze cephalosporins with an oxyimino side chain (Malloy and Campos, 2011). Some ESBL genes are mutant derivatives of established plasmid-mediated β-lactamases (e.g., *bla*_TEM/SHV_) or mobilized from environmental bacteria (e.g., *bla*_CTX–M_) (Overdevest et al., 2011). Many Enterobacteriaceae now carry ESBL genes such as *bla*_CTX–M_, with particular genes associated with different geographical regions (Hawkey and Jones, 2009).

It is generally assumed that antibiotics can enhance HGT, and also a substantial amount of data suggest that sub-inhibitory concentrations of antibiotics may significantly increase the conjugation transfer frequency both *in vitro* and in the animal gut (Aminov, 2011). For instance, sub-inhibitory concentrations of β-lactams and tetracycline enhanced the conjugative transfer of a tetracycline resistance plasmid in *Staphylococcus aureus* or the conjugative transposon CTnDOT in *Bacteroides* cells, respectively (Barr et al., 1986; Stevens et al., 1993; Whittle et al., 2002). The presence of tetracycline or erythromycin increased the transfer frequency of conjugative transposon Tn*916* or plasmid pLFE1 in the guts of gnotobiotic rats, respectively (Bahl et al., 2004; Feld et al., 2008), and exposure to gentamicin resulted in a significant increase of conjugation transfer of a mobilizable gentamicin resistance plasmid pUCP24T in an *E. coli* SM10λπ-*Pseudomonas aeruginosa* PAO1 conjugation model (Lu et al., 2017). Furthermore, ciprofloxacin treatment has been shown to enhance the transfer of the ICE SXT, which confer some antibiotic resistance phenotypes in Enterobacteriaceae (Beaber et al., 2004). However, it has been pointed out, that in many conjugation setups, it is not possible to distinguish between the effects of the antibiotic on conjugation efficiency and its effect on selection dynamics, and that antibiotics may only serve as a selective driver for conjugation dynamics (Lopatkin et al., 2016).

In a previous study, we designed an experimental setup for measurement of conjugation frequency in which we could separate conjugation rate from the power of selection by the antibiotic by pre-growing the donor in the presence of [1/2 minimal inhibitory concentration (MIC)] of antibiotics and performing the conjugation in an antibiotic-free environment for 30 or 60 min (Moller et al., 2017). We showed that the transfer frequency of a naturally occurring *bla*_CTX–M–__1_ encoding resistance plasmid, pTF2, transferred into *E. coli* MG1655, was enhanced significantly when the donor was pre-grown in broth containing CTX, and that the enhanced transfer happened in a SOS-independent manner (Moller et al., 2017). Using our newly developed experimental setup, we aimed to investigate whether this antibiotic-induced plasmid transfer (PT) also applies to other plasmid types harbored in their natural host strains, and whether it applied to other classes of antibiotics. Our findings reveal that different antibiotics are able to induce increased conjugation transfer in different *E. coli* strains with various ESBL encoding plasmids.

## Materials and Methods

### Bacterial Strains

Twenty-five ESBL-producing *E. coli* strains collected in Denmark in 2015 were included in the study. These isolates were collected and whole genome sequenced (WGS) within the Danish programme for surveillance of antimicrobial resistance (DANMAP) 2015 (see Supplementary Table 1 for ENA accession information: PRJEB14641 and PRJEB14086), and originated from pigs’ cecal samples (*n* = 13), calves’ cecal samples (*n* = 6), and chicken meat (*n* = 6) (Table 1). They were selected among the animal- and food-origin ESBL-producing *E. coli* from DANMAP 2015 to include all detected ESBL genes and all possible plasmids harboring such genes based on a rapid preliminary analysis of beta-lactamase-encoding genes and plasmid replicons detected by using the Center for Genomic Epidemiology (CGE)^1^ tools. *E. coli* K-12 MG1655/pTF2 (Kjeldsen et al., 2015) with the IncI1 plasmid pTF2 carrying *bla*_CTX–M–__1_ (IncI1/pST49/CTX-M-1) was also used in this study. *E. coli* J53-1 (Nal^R^) (Taylor and Levine, 1980) and *E. coli* J53-2 (Rif^R^) (Appelbaum et al., 1972) were used as recipients in conjugation experiments.

**TABLE 1:** Genetics features of commensal *E. coli* isolates based on whole genome sequence analysis.

**Isolate name^a^**	**β-Lactamase (*bla*-type)**	**MLST type**	**Plasmid replicons^b^**	**pMLST^d^**	**Antimicrobial resistance genes^k^**	**Virulence genes**
ESBL 1	CTX-M-1	ST101	IncI1	3	*dfr*A17, *aad*A1, *aad*A5, *bla*_CTX–M–__1_, *sul*1, *sul*2, *tet*(A), *cat*A1^l^	*gad*, *iroN*, *iss*, *lpfA*, *mchB*, *mchC*, *mchF*, *mcmA*
ESBL 2	CTX-M-1	ST4373	IncFII, IncFIB(AP001918)	–	*dfr*A14, *str*A^l^, *str*B, *bla*_CTX–M–__1_, *bla*_TEM–__1__A_, *sul*2, *tet*(B)	*air*, *eilA*, *gad*, *iroN*, *lpfA*
ESBL 3	CTX-M-1	ST4580	IncI1, ColpVC, pO111	7	*dfrA*1, *aadA*1, *bla*_CTX–M–__1_, *sul*1^l^, *sul*2	*cnf1*, *gad*, *iroN*, *iss*, *lpfA*, *mchB*, *mchC*, *mchF*, *mcmA*
ESBL 4	CTX-M-1	ST58	IncI1	7	*dfr*A1, *aad*A1, *bla*_CTX–M–__1_, *sul*1^l^	*gad*, *iss*, *lpfA*
ESBL 5	CTX-M-1	ST165	IncFII (pRSB107), pO111	–	*bla*_CTX–M–__1_, *tet*(A), *erm*(B)^l^, *mph*(A)	*gad*
ESBL 6	CTX-M-1	ST295	IncFIC (FII), IncFIB(AP001918)	–	*dfr*A8, *aad*A1, *bla*_CTX–M–__1_, *mph*(A)	*iroN*, *iss*, *lpfA*, *mchF*, *tsh*
ESBL 7	CTX-M-1	ST362	IncFII, IncI1, IncQ1**^c^**	NT**^e^**	*dfr*A1, *dfr*A17, *aph*(3′)-Ia, *str*B^l^, *aph*(4)-Ia, *aac*(3)-IVa, *aad*A1, *str*A, *aad*A5, *bla*_CTX–M–__1_, *bla*_TEM–__1__B_, *sul*2, *tet*(A), *cat*A1	*ireA*, *iss*, *mcmA*
ESBL 8	CTX-M-1	ST345	IncI1, IncFIB(AP001918), Col156	3	*dfr*A17, *str*B^l^, *aad*A5, *bla*_CTX–M–__1_, *bla*_TEM–__1__C_, *sul*2, *tet*(A)	*cnf1*, *gad*, *iroN*, *iss*, *lpfA*, *mchF*
ESBL 9	CTX-M-1	ST101	IncFII(29), IncI1	NT**^e^**	*dfr*A17, *aad*A1, *aad*A5, *bla*_CTX–M–__1_, *sul*1, *sul*2, *tet*(A), *cat*A1^l^	*cba*, *cma*, *gad*, *iroN*, *iss*, *lpfA*, *mchB*, *mchC*, *mchF*, *mcmA*
ESBL 10	CTX-M-1	ST647	IncFII(pSE11), IncI1, IncFIB(AP001918)	293**^f^**	*bla*_CTX–M–__1_	*cba*, *cdtB*, *cma*, *gad*, *iroN*, *iss*, *sfaS*, *vat*
ESBL 11	CTX-M-1	ST48	IncFII(29), IncI1, IncY, IncFIB(AP001918), Col(BS512), Col(MP18)	294**^g^**	*aad*A1, *bla*_CTX–M–__1_, *sul*2	ND^m^
ESBL 12	CTX-M-1	ST1640	IncFII, IncI1, IncN, IncFIB(AP001918), IncQ1**^c^**, ColpVC	295**^h^**	*dfr*A14^l^, *dfr*A17, *qnr*S1, *gyr*A S83L, *str*B^l^, *str*A^l^, *aad*A5, *bla*_CTX–M–__1_, *bla*_TEM–__1__B_, *sul*2, *tet*(A), *cat*A1^l^	*astA*, *cba*, *celb*, *cma*, *gad*, *hlyE*, *iha*, *ireA*, *iss*
ESBL 13	CTX-M-1	ST4243	IncI1 **^c^**, ColpVC, IncB/O/K/Z	NT**^i^**	*dfr*A1, *bla*_CTX–M–__1_, *bla*_TEM–__1__B_, *sul*2	*air*, *astA*, *cma*, *eilA*, *gad*, *iha*
ESBL 14	CTX-M-1	ST117	IncFII, IncI1, IncFIB(AP001918), ColpVC	296**^j^**	*dfr*A17, *aad*A5, *sul*2, *bla*_CTX–M–__1_	*gad*, *hlyE*, *ireA*, *iss*, *lpfA*, *vat*, *mchB*, *mchC*, *mchF*, *mcmA*
ESBL 15	CTX-M-1	ST752	IncFIC(FII), IncI1, IncFIB(AP001918), pO111	7	*str*A, *str*B, *bla*_CTX–M–__1_	*astA*, *cif*, *eae*, *espA*, *espB*, *espF*, *gad*, *iroN*, *iss*, *nleB*, *tccP*, *tir*, *tsh*
ESBL 16	CTX-M-1	ST88	IncFII, IncI1, Col(MG828), IncFIB(AP001918)	7	*dfr*A1, *str*A, *str*B, *aad*A1, *bla*_CTX–M–__1_, *bla*_TEM–__1__C_, *sul*1^l^, *sul*2, *tet*(A)	*gad*, *iroN*, *iss*, *lpfA*, *mchF*
ESBL 17	CTX-M-1	ST101	IncFII, IncI1, IncFIB(AP001918)	3	*dfr*A5, *dfr*A17, *aad*A5, *bla*_CTX–M–__1_, *sul*2	*gad*, *iroN*, *iss*, *lpfA*, *mchF*
ESBL 18	TEM-52	ST10	IncFIA, IncFII, IncFIB(AP001918), IncX1	–	*bla*_TEM–__52__B_	*astA*, *gad*, *iss*
ESBL 19	TEM-52	ST10	IncFIA, IncFII, IncX1, IncFIB(AP001918)	–	*bla*_TEM–__52__B_	*astA*, *gad*, *iss*
ESBL 20	TEM-52	ST2607	IncFIC(FII), IncI1, IncN, IncFIB(AP001918)	–	*aad*A17^l^, *bla*_TEM–__52__B_	*gad*, *iss*, *lpfA*
ESBL 21	CTX-M-55	ST2952	IncI1, IncX1	16	*bla*_CTX–M–__55_	*gad*, *lpfA*
ESBL 22	CTX-M-14	ST1310	IncFII(29), IncFIA, IncFIB(AP001918)	–	*bla*_CTX–M–__14_	*gad*, *iss*, *lpfA*
ESBL 23	CTX-M-14	ST88	IncFII, IncFIB(AP001918), IncB/O/K/Z, IncQ1**^c^**	–	*str*A, *str*B, *bla*_CTX–M–__14_, *bla*_TEM–__1__B_, *sul*2, *tet*(A)	*cnf1*, *gad*, *iroN*, *iss*, *lpfA*, *mchF*
ESBL 24	CTX-M-14	ST34	IncI1, IncQ1**^c^**, Col(MG828), ColpVC	259	*qnr*B19^l^, *str*A, *str*B^l^, *bla*_CTX–M–__14_, *bla*_TEM–__1__B_, *sul*2, *tet*(A), *flo*R^l^	ND**^m^**
ESBL 25	SHV-12	ST115	IncFII, IncFIB(AP001918), pO111	–	*qnr*B19, *bla*_SHV–__12_	*air*, *celb*, *eilA*, *iha*, *iss*

*^*a*^Original ID as recorded in European Nucleotide Archive (ENA) is provided in Supplementary Table 1. Isolates for which ESBL plasmid transfer significantly increased upon antibiotic exposure are underlined. ^*b*^The replicons of the plasmids associated with the ESBL gene are underlined. ^*c*^Only part of the replicon was detected. ^*d*^pMLST was performed on IncI1. ^*e*^No detection of the *sogS* allele. The remaining allele variants were *repI1*, 2; *ardA*, 1; *trbA*, 4; *pilL*, 2. ^*f*^New pMLST identified in this study. The allele variants were *repI1*, 3; *ardA*, 4; *trbA*, 13; *sogS*, 3; *pilL*, 25. ^*g*^New pMLST identified in this study. The allele variants were *repI1*, 2; *ardA*, 14; *trbA*, 5; *sogS*, 4; *pilL*, 2. ^*h*^New pMLST identified in this study. The allele variants were *repI1*, 2; *ardA*, 48; *trbA*, 4; *sogS*, 1; *pilL*, 2. ^*i*^No detection of both the *trbA* and the *sogS* alleles. The remaining allele variants were *repI1*, 10; *ardA*, 6; *pilL*, 2. ^*j*^New pMLST identified in this study. The allele variants were *repI1*, 2; *ardA*, 49; *trbA*, 4; sogS, 1; *pilL*, 2. ^*k*^Antimicrobial resistance phenotype: aminoglycoside [*aad*A1, *aad*A5, *aad*A17, *str*A, *str*B, *aph*(3′)-Ia, *aph*(4)-Ia, *aac*(3)-IVa], quinolone (*gyr*A S83L, *qnr*S1, *qnr*B19), trimethoprim (*dfr*A1, *dfr*A5, *dfr*A8, *dfr*A14, *dfr*A17), beta-lactam (*bla*_*CTX–M–*__1_, *bla*_*CTX–M–*__14_, *bla*_*CTX–M–*__55_, *bla*_*TEM–*__1__*A*_, *bla*_*TEM–*__1__*B*_, *bla*_*TEM–*__1__*C*_, *bla*_*TEM–*__52__*B*_, *bla*_*SHV–*__12_), sulfonamide (*sul*1, *sul*2), tetracycline (*tet*A, *tet*B), macrolide (*mph*A, *erm*B), and phenicol (*cat*A1, *flo*R). ^*l*^Genes with <100% match with the reference in ResFinder database. ^*m*^ND, not identified.*

### Bioinformatics Analysis

Sequencing reads were quality trimmed using Trimmomatic-v0.32 (Bolger et al., 2014) and *de novo* assembled using SPAdes v.3.5.0 (Bankevich et al., 2012). The assembled genomes were analyzed via the CGE (see text footnote 1) webserver by using a bacterial analysis pipeline (BAP) (Thomsen et al., 2016). Multi-locus sequence types were determined using MLST 2.0 with the allele sequence and profile data from PubMLST^2^. Other genotypic features such as plasmid replicons, antimicrobial resistance genes, and virulence genes were also identified with a selected threshold equal to 98% identity using the pipelines, PlasmidFinder 2.0, VirulenceFinder 2.0, and ResFinder 3.2 at CGE. The sequences were analyzed for the presence or absence of *tra* genes, using BLAST in CLC Main Workbench 8.0.0 (CLC Bio, Denmark). To reconstruct ESBL plasmids, a curated dataset of complete plasmid sequences (Orlek et al., 2017) was used to identify the best reference plasmid for each strain using (i) information on replicon, pMLST, and ESBL gene content; and (ii) highest percentage of ID over highest percentage of length as obtained using nucleotide BLAST at NCBI. Using the best reference plasmid, contigs were assigned to a plasmid scaffold within each strain using nucleotide BLAST at NCBI.

### Antibiotic Susceptibility Testing

The MIC of CTX and AMP was determined on donors and transconjugants from the first conjugation round (see below) using the broth microdilution method [concentrations ranging from 0 to 2048 mg L^–1^ (CTX) or 0 to 4096 mg L^–1^ (AMP) by twofold dilution increases] in poly-styrene microtiter plates using the control strain *E. coli* ATCC^®^ 25922 according to the CLSI guidelines M100-S25 (Wayne et al., 2015). In addition, MIC determination experiments were performed using serial increases of 200 mg L^–1^ AMP to obtain a value as close to the real MIC as possible. The MIC of CIP was measured by using ciprofloxacin single pack *E*-test (Biomerieux, United States) (concentration ranging from 0.002 to 32 mg L^–1^) following the manufacturer’s protocol. MIC values for donors and transconjugants are available in Supplementary Table 2.

### Conjugation Experiment

The 25 strains containing different ESBL plasmids and MG1655/pTF2 (Kjeldsen et al., 2015) were used as donor strains and *E. coli* J53-2 as recipient strain in the first round of conjugation. In the second round, transconjugants from the first round of conjugation were used as donors and *E. coli* J53-1 as recipient. Strains were exposed to 1/2 MIC of antibiotics. Conjugation was performed as previously described (Moller et al., 2017). Briefly, donor strains were grown either without or with sub-inhibitory concentrations (1/2 MIC) of antibiotics in Luria-Bertani (LB) broth (Sigma, Copenhagen, Denmark) with shaking (125 rpm) at 37°C to exponential phase (OD_600_ = 0.5), and the recipient strain was grown without antibiotics to the same OD_600_. Antibiotics were removed by a washing step and conjugation was performed by mixing donor and recipient strain in a 1:1 ratio on filters (0.22 μM, Millipore, Copenhagen, Denmark) on LB agar plates (Becton, Dickinson, Albertslund, Denmark) at 37°C for 30 and 60 min. The bacterial material was washed from the filters using isotonic NaCl and plated on LB agar plates containing (in the first conjugation round): 2 mg L^–1^ CTX (for quantifying donor + transconjugants) or 50 mg L^–1^ rifampicin and 2 mg L^–1^ CTX (quantifying transconjugants only), or grown on LB agar plates containing (in the second conjugation round): 2 mg L^–1^ CTX and 50 mg L^–1^ rifampicin (quantifying donor) or 10 mg L^–1^ nalidixic acid and 2 mg L^–1^ CTX (quantifying transconjugants only), and incubated overnight at 37°C. The conjugation frequency was calculated as the number of transconjugants divided by the number of donors. The conjugation experiments were performed in three biological replicates with three technical replicates each.

### Plasmid Profiling and PCR-Based Replicon Typing

Transconjugants from the first conjugation round showing significant antibiotic-induced increase in PT and their respective donors were selected for plasmid profiling. Plasmid DNA was purified according to the method described by Kado and Liu (1981), followed by separation on a 0.7% (W/V) agarose gel for 4 h at 150 V and stained with GelRed (Biotium, Hayward, CA, United States). The approximate molecular weight of each plasmid was determined by comparison with two reference *E. coli* strains, 39R861 and V517, containing multiple reference plasmids (Macrina et al., 1978; Karczmarczyk et al., 2011).

The plasmid replicon types of the ESBL plasmids were confirmed in the transconjugants from the first conjugation round using PCR-Based Replicon Typing (PBRT) 2.0 Kit (Diatheva, Fano, Italy). The total DNA was obtained by boiling lysis method exposing cells to 100°C for 10 min, and clarifying the lysate preparations by centrifugation at 15,000 × *g* for 10 min. One microliter of the supernatant was tested in PCR assays and the amplification products were analyzed by 2.5% agarose gel electrophoresis as described previously (Carattoli et al., 2005; Carloni et al., 2017).

### Plasmid Sequencing

Plasmid DNA was isolated from the transconjugants from the first conjugation round showing significant antibiotic-induced increase in PT using PureLink HiPure Plasmid DNA Purification Kits (Invitrogen, CA, United States), and the plasmid DNA was subjected to pair-end-read format using the Nextera XT DNA Library Preparation Kit and sequenced in an Illumina MiSeq (Illumina, Inc., San Diego, CA, United States) essentially as previously described (McElhoe et al., 2014). Obtained sequences were analyzed by PlasmidFinder and BLAST in CLC Main Workbench 8.0.0 (CLC Bio, Denmark) to confirm presence of the ESBL plasmids.

### RNA Extraction

Single colonies of transconjugants from the first conjugation round were grown in LB broth with shaking (125 rpm) overnight at 37°C. The cultures were diluted 1000-fold and grown with and without antibiotics to OD_600_ = 0.5. The antibiotic concentration was 1/2 MIC. A FastPrep cell disrupter system (Qbiogene, Illkirch, France) and RNeasy Mini Kit (Qiagen, Sollentuna, Sweden) were used to extract total RNA by mechanical disruption. Quantity of the extracted RNA was determined by A260 measurements and purity by A260/280 ratio measurements using a NanoDrop 1000 spectrophotometer (Thermo Scientific, Hvidovre, Denmark). RNA (1 μg) samples were purified by DNA digestion using TURBO^TM^ DNase kit (2 U/μL) (Ambion, Life Technologies, Nærum, Denmark) to remove contaminating genomic DNA.

### Reverse-Transcribed-Quantitative Real Time Polymerase Chain Reaction

RNA was reverse-transcribed into cDNA using the High Capacity cDNA Reverse Transcription Kit (Life Technologies, Nærum, Denmark). Reverse-transcribed-quantitative real time polymerase chain reaction (RT-qPCR) was performed using FastStart Essential DNA Green Master (Roche, Hvidovre, Denmark) and a LightCycler 96 (Roche, Hvidovre, Denmark). Expression levels of *traF*, *traI*, *traL*, *traM*, *pilS*, *recA*, and *sfiA* were measured (primer sequences, Supplementary Table 3). The expression data were normalized to two validated reference genes, *gapA* and *nusG* (Kjeldsen et al., 2015). RT-qPCR was performed on two biological replicates with two technical replicates each, and the results were calculated by the 2^–ΔΔ*Ct*^ method (Pfaffl, 2001).

### Statistical Analysis

Statistical analysis used in this study was performed using GraphPad Prism version 7.03. Data are expressed as the means ± standard deviation (SD) from at least three independent experiments. Comparison of gene expression and conjugation frequencies with and without antibiotics were analyzed using Student’s *t*-test with Welch’s correction. *P*-values ≤ 0.05 were considered statistically significant.

## Results

### Analysis of 25 *E. coli* Strains and Their Plasmids

With the aim of investigating PT of different plasmid types in various *E. coli* strains, we obtained 25 ESBL-producing *E. coli* strains isolated from pigs, calves, and chicken meat. A whole genome sequence analysis was undertaken, and a total of 19 MLST types were identified among the *E. coli* strains (Table 1). Of the 25 strains, 17 carried *bla*_CTX–M–__1_, 3 *bla*_TEM–__52__B_, 3 *bla*_CTX–M–__14_, 1 *bla*_CTX–M–__55_, and 1 *bla*_SHV–__12_. In 20 strains, genes conferring resistance to additional non-beta-lactam antibiotics were detected. Most (*n* = 23) strains harbored multiple plasmid replicons (Table 1). Plasmid multilocus sequence typing (pMLST) was performed on IncI1 plasmids, leading to identification of eight pMLST types including four newly described in this study (pST293-296) that were submitted to the Plasmid MLST Databases^3^. Three IncI1 plasmids were untypeable as one or two alleles were not detected (Table 1).

### Antibiotics Increase Conjugation Transfer Frequency

Conjugation transfer frequency was investigated using the 25 diverse strains and the previously investigated MG1655/pTF2 was included as positive control (Moller et al., 2017). PT was affected by antibiotic exposure in 8 (30.8%) out of the 26 strains. These carried IncI1/pST7/CTX-M-1 (ESBL 4), IncI1/pST49/CTX-M-1 (MG1655/pTF2), IncI1/pST293/CTX-M-1 (ESBL 10), IncI1/pST295/CTX-M-1 (ESBL 12), IncI1/pST3/CTX-M-1 (ESBL 17), IncI1/pST16/CTX-M-55 (ESBL 21), and IncFII/CTX-M-14 (*n* = 2, ESBL 22 and ESBL 23) (Table 2). Pre-growth with 1/2 MIC of CTX increased the PT significantly for all of these eight strains after both 30 and 60 min of cell contact compared to conjugation experiments in which the donor was grown without CTX before the conjugation. Additionally, growth with 1/2 MIC of AMP increased the PT significantly in six of these eight strains (MG1655/pTF2, ESBL 4, 10, 12, 21, and 22) while CIP treatment only increased the PT significantly in three of the eight strains (MG1655/pTF2, ESBL 4, and ESBL 22). A non-significant CIP-induced PT was observed for three other strains out of the eight (ESBL 10, 12, and 21) (Table 2). We obtained transconjugants from eight other strains (ESBL 1, 5, 6, 8, 11, 14, 16, and 18), but observed no significant antibiotic induced PT using any of the three antibiotics (Supplementary Figure 1). We did not obtain any transconjugants either without or with 1/2 MIC of AMP, CTX, or CIP exposure for the remaining 7 *bla*_CTX–M_-positive, 2 *bla*_TEM–__52__B_-positive, and 1 *bla*_SHV–__12_-positive strains used in this study (ESBL 2, 3, 7, 9, 13, 15, 19, 20, 24, and 25). When analyzing these 10 strains from which no transconjugants were observed, the *traF* gene was absent in 7 strains (ESBL 2, 7, 9, 13, 19, 20, and 25), and *traI*, *traL*, and *traM* lacked in ESBL 19. The transconjugants were named after the ESBL number of the original donor, followed by the antibiotic used in the conjugation experiment.

**TABLE 2:** Fold changes of antibiotic (CTX, AMP, or CIP)-induced increased conjugation transfer frequency using ESBL *E. coli* as donors and J53-2 as recipient.

**Donor**	**Conjugation time (min)**	**Conjugation transfer frequency^a^ (Control)**	**Conjugation transfer frequency^a^ (CTX)**	**Fold change^b^**	**Conjugation transfer frequency^a^ (AMP)**	**Fold change^b^**	**Conjugation transfer frequency^a^ (CIP)**	**Fold change^b^**
MG1655/pTF2 (IncI1/CTX-M-1)	30	4.63 × 10^–3^ ± 1.25 × 10^–3^	2.93 × 10^–2^ ± 0.23 × 10^–2^	6.3^∗∗^	1.30 × 10^–2^ ± 0.11 × 10^–3^	2.8^∗^	5.73 × 10^–3^ ± 0.80 × 10^–3^	1.2
	60	1.11 × 10^–2^ ± 0.24 × 10^–2^	4.09 × 10^–2^ ± 0.24 × 10^–2^	3.7^∗∗^	6.11 × 10^–2^ ± 0.51 × 10^–2^	5.5^∗^	6.97 × 10^–2^ ± 1.04 × 10^–2^	6.3^∗^
ESBL 4 (IncI1/CTX-M-1)	30	4.11 × 10^–4^ ± 0.73 × 10^–4^	1.04 × 10^–3^ ± 0.39 × 10^–3^	2.5	3.63 × 10^–3^ ± 1.13 × 10^–3^	8.8	1.39 × 10^–3^ ± 0.33 × 10^–3^	4.7
	60	5.71 × 10^–4^ ± 0.74 × 10^–4^	1.9 × 10^–3^ ± 0.38 × 10^–3^	3.3^∗^	6.53 × 10^–3^ ± 0.67 × 10^–3^	11.3^∗^	4.67 × 10^–3^ ± 0.70 × 10^–3^	8.1^∗^
ESBL 10 (IncI1/CTX-M-1)	30	1.04 × 10^–2^ ± 0.12 × 10^–2^	3.62 × 10^–2^ ± 1.22 × 10^–2^	3.5	2.93 × 10^–2^ ± 0.28 × 10^–2^	2.8^∗^	2.44 × 10^–2^ ± 0.58 × 10^–2^	2.3
	60	1.17 × 10^–2^ ± 0.11 × 10^–2^	1.02 × 10^–1^ ± 0.11 × 10^–1^	8.7^∗^	2.97 × 10^–2^ ± 0.31 × 10^–2^	2.5^∗^	5.26 × 10^–2^ ± 1.21 × 10^–2^	4.5
ESBL 12 (IncI1/CTX-M-1)	30	8.46 × 10^–5^ ± 1.69 × 10^–5^	2.44 × 10^–4^ ± 0.16 × 10^–4^	2.9^∗^	2.85 × 10^–4^ ± 0.55 × 10^–4^	3.3	1.47 × 10^–4^ ± 0.19 × 10^–4^	1.7
	60	1.47 × 10^–4^ ± 0.09 × 10^–4^	1.77 × 10^–3^ ± 0.38 × 10^–3^	12.0^∗^	6.63 × 10^–4^ ± 1.24 × 10^–4^	4.5^∗^	5.61 × 10^–4^ ± 1.42 × 10^–4^	3.8
ESBL 17 (IncI1/CTX-M-1)	30	2.63 × 10^–5^ ± 0.33 × 10^–5^	5.61 × 10^–5^ ± 0.72 × 10^–5^	2.1^∗^	3.35 × 10^–5^ ± 1.41 × 10^–5^	1.2	3.20 × 10^–5^ ± 1.41 × 10^–5^	1.2
	60	4.15 × 10^–5^ ± 1.07 × 10^–5^	1.09 × 10^–4^ ± 0.20 × 10^–4^	2.6^∗^	4.20 × 10^–5^ ± 1.54 × 10^–5^	1.0	3.29 × 10^–5^ ± 0.95 × 10^–5^	0.8
ESBL 21 (IncI1/CTX-M-55)	30	2.73 × 10^–3^ ± 0.40 × 10^–3^	8.96 × 10^–3^ ± 1.99 × 10^–3^	3.3	8.63 × 10^–3^ ± 2.03 × 10^–3^	3.2	9.34 × 10^–3^ ± 0.66 × 10^–3^	3.4
	60	5.41 × 10^–3^ ± 0.18 × 10^–2^	1.62 × 10^–2^ ± 0.27 × 10^–2^	3.3^∗^	1.45 × 10^–2^ ± 0.11 × 10^–3^	2.7^∗^	1.30 × 10^–2^ ± 0.15 × 10^–2^	2.4
ESBL 22 (IncF/CTX-M-14)	30	1.61 × 10^–4^ ± 0.36 × 10^–4^	5.91 × 10^–4^ ± 1.18 × 10^–4^	3.7^∗^	9.3 × 10^–4^ ± 0.95 × 10^–4^	5.8^∗^	9.67 × 10^–4^ ± 0.94 × 10^–4^	6.0^∗^
	60	2.72 × 10^–4^ ± 0.94 × 10^–4^	8.69 × 10^–4^ ± 2.26 × 10^–4^	3.2	1.37 × 10^–3^ ± 0.14 × 10^–3^	5.0^∗^	1.22 × 10^–3^ ± 0.19 × 10^–3^	4.5^∗^
ESBL 23 (IncF/CTX-M-14)	30	2.70 × 10^–4^ ± 0.17 × 10^–4^	7.35 × 10^–4^ ± 0.55 × 10^–4^	2.7^∗^	3.11 × 10^–4^ ± 0.28 × 10^–4^	0.9	2.87 × 10^–4^ ± 0.22 × 10^–4^	1.1
	60	3.98 × 10^–4^ ± 0.70 × 10^–4^	1.07 × 10^–3^ ± 0.18 × 10^–3^	2.7^∗^	3.79 × 10^–4^ ± 0.55 × 10^–4^	1.0	4.28 × 10^–4^ ± 0.35 × 10^–4^	1.1

*^*a*^The results shown are from two biological replicates with three technical replicates each, the data shown represent the mean ± standard deviation. ^*b*^The asterisks indicate statistical significance between antibiotic treated and control at level: ^∗^*P* ≤ 0.05, ^∗∗^*P* ≤ 0.01.*

Plasmid profiling and PBRT and sequencing of the transconjugants from the first conjugation round confirmed the presence of the replicons of the ESBL plasmids identified by WGS data analysis and allowed further insights into the IncI1 replicons. PBRT revealed that IncI1α and IncI1γ replicons co-existed in two transconjugants, namely 10AMP and 10CIP (transconjugant originating from ESBL10 treated with AMP or CIP, respectively). The occurrence of both replicons was verified in the donor (ESBL10) and transconjugants by analysis of the WGS data, and was also confirmed by plasmid profiling (data not shown). It is interesting to note that in transconjugant 10CTX, only the IncI1α replicon was transferred. An additional case in which the transconjugants harbored other plasmids besides the ESBL plasmid was represented by 12AMP, which harbored a non-typeable plasmid and a Col156 plasmid besides the IncI1α ESBL encoding plasmid. In all remaining cases, the transconjugants harbored only the ESBL encoding plasmids which were either IncI1α (4CTX, 12CTX, 12AMP, 17CTX, 17AMP, 17CIP, 21CTX, 21AMP, and 21CIP) or IncFII (22CTX, 22AMP, 22CIP, 23CTX, 23AMP, and 23CIP).

### Expression of *tra* Genes Is Induced by Antibiotics in the Transconjugants

**FIGURE 1:**
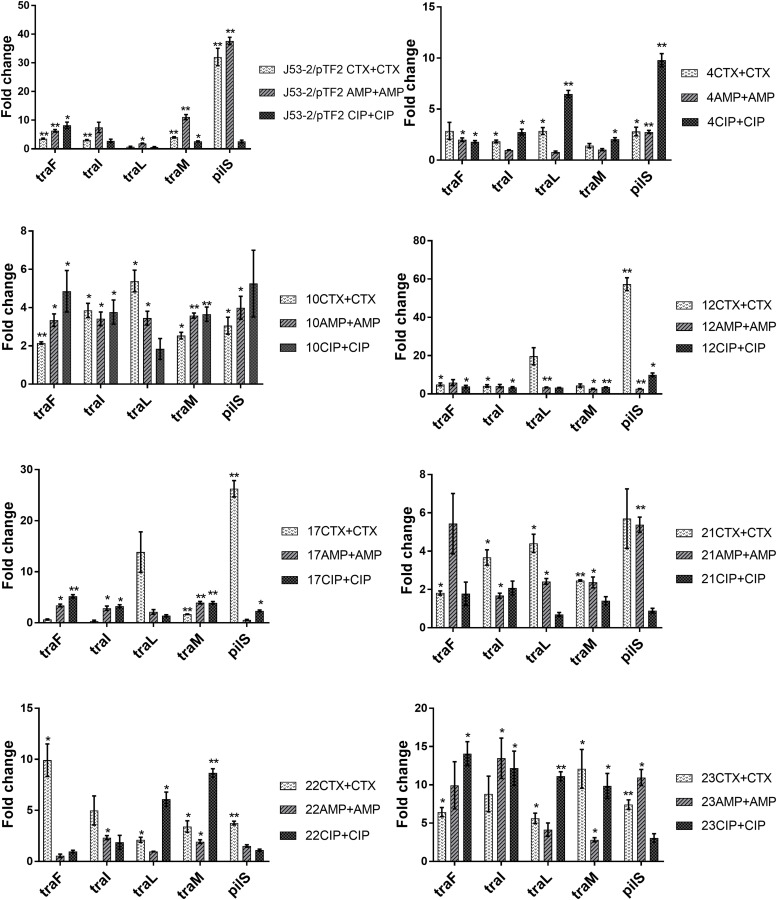
Expression of genes involved in conjugation in the transconjugants from the first round of conjugation. Cell samples grown with no or 1/2 MIC of antibiotics (+CTX, AMP, or CIP) were used for RT-qPCR. Data are presented as fold change relative to control without antibiotics. The combination of strain number with antibiotic name represents the transconjugants from the first round of conjugation obtained after exposure to the specified antibiotic. The expression data were normalized to the reference genes, *gapA* and *nusG*. The results shown are means of three biological replicates with two technical replicates each and the error bars represent standard errors of the means. The stars indicate statistical significance at different levels: ^∗^*P* ≤ 0.05, ^∗∗^*P* ≤ 0.01.

Previous data had shown that increased PT was linked to up-regulation of the transfer (*tra*) genes (Moller et al., 2017). In order to investigate whether the *tra* genes on the conjugative ESBL encoding plasmids able to transfer were up-regulated in response to exposure to antibiotics, we exposed the transconjugants from the first round of conjugation to the three antibiotics at a concentration of 1/2 MIC. We observed that several *tra* genes were significantly up-regulated in all of the eight transconjugants when exposed to CTX, AMP, and CIP (Figure 1). To rule out that the observed increase in expression levels was caused by antibiotic induced increases in plasmid copy number, we performed RT-qPCR. We found that the ratio between a plasmid encoded gene (*traF*) and a chromosomally encoded gene (*dxs*) did not change significantly due to antibiotic exposure, supporting that the antibiotics do not affect copy-number (Supplementary Figure 2).

**FIGURE 2:**
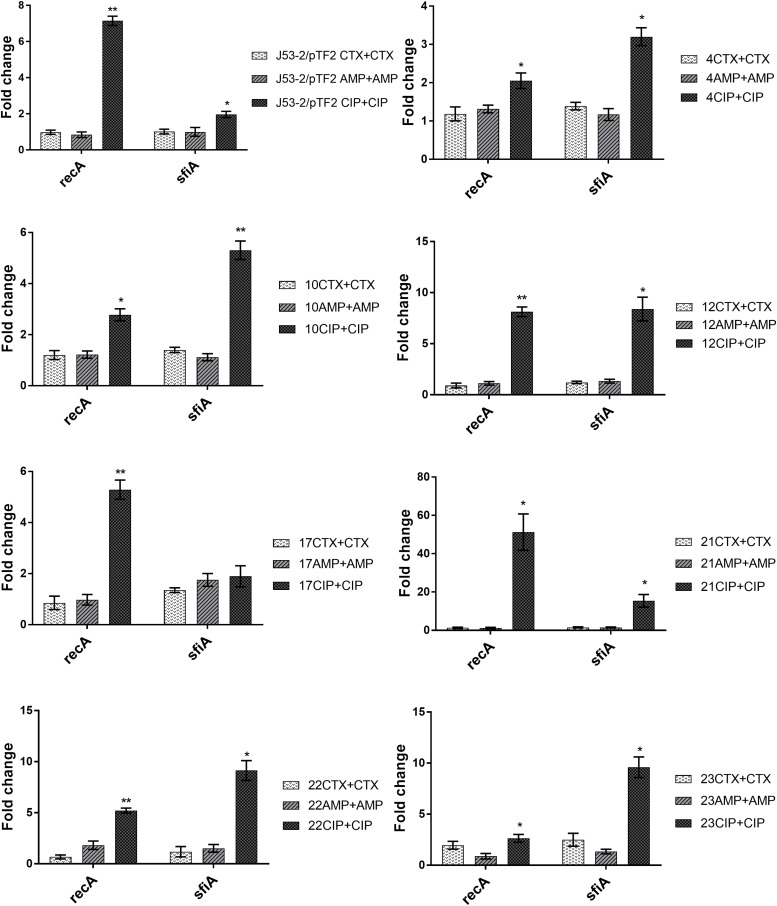
Expression of SOS-responsive genes in the transconjugants from the first round of conjugation. Cell samples grown with no or 1/2 MIC of antibiotics (+CTX, AMP, or CIP) were used for RT-qPCR. Data are presented as fold change relative to control without antibiotics. Note that the different figures are not drawn to the same scale. The combination of strain number with antibiotic name represents the transconjugants from the first round obtained after exposure to the specified antibiotic. The expression data were normalized to two validated reference genes, *gapA* and *nusG*. The results shown are means of three biological replicates with two technical replicates each and the error bars represent standard errors of the means. The stars indicate statistical significance at different levels: ^∗^*P* ≤ 0.05, ^∗∗^*P* ≤ 0.01.

To investigate the involvement of the SOS response in antibiotic induced PT, we analyzed the expression of *recA* and *sfiA*. RT-qPCR analysis showed that expression of these two genes was not affected significantly by growth in the presence of CTX and AMP (Figure 2). In contrast, CIP treatment increased the expression of *recA* significantly in all of these eight strains, and increased the level of *sfiA* significantly in seven of these strains (Figure 2).

### Antibiotics Induce Conjugation From Transconjugants

In order to investigate whether the up-regulated *tra* genes in transconjugants from the first round of conjugation would also lead to increased PT frequency, a second conjugation experiment was performed using the transconjugants as donors and *E. coli* J53-1 as recipient strain. The experiment showed that the CTX treatment increased significantly PT of IncI1/pST7/CTX-M-1 plasmid (4CTX), IncI1/pST293/CTX-M-1(10CTX), IncI1/pST295/CTX-M-1 (12CTX), IncI1/pST3/CTX-M-1 (17CTX), IncI1/pST16/CTX-M-55 (21CTX), and IncF/CTX-M-14 plasmids (22CTX), compared to conjugation without pre-growth in the presence of CTX (Table 3). The AMP treatment also showed significantly increased PT in IncI1/pST7/CTX-M-1 plasmid (4AMP) and IncI1/pST3/CTX-M-1 (17AMP). Additionally, CIP treatment increased significantly PT of IncI1/pST7/CTX-M-1 plasmid (4CIP), IncI1/pST3/CTX-M-1 (17CIP), IncI1/pST16/CTX-M-55 (21CIP), and IncF/CTX-M-14 plasmids (22CIP). For the two remaining plasmids we observed a, non-significant, increased antibiotic induced PT (Table 3).

**TABLE 3:** Fold changes of antibiotic-induced increased conjugation transfer frequency using transconjugants from first conjugation round as donors and J53-1 as recipient.

**Conjugation time (min)**	**Donor**	**PT difference (CTX)**	**Donor**	**PT difference (AMP)**	**Donor**	**PT difference (CIP)**
30	MG1655/pTF2 CTX	1.6	MG 1655/pTF2 AMP	1.6	MG1655/pTF2 CIP	1.3
60		1.6		2.2		3.1
30	4CTX	28.9^∗∗^	4 AMP	6.3^∗^	4CIP	7.6^∗^
60		18.7^∗∗^		3.7		3.7^∗∗^
30	10CTX	8.6^∗^	10 AMP	3.3	10CIP	6.8
60		3.0^∗^		1.3		2.6
30	12CTX	3.1	12 AMP	2.6	12CIP	2.0
60		2.2^∗^		1.8		1.6
30	17CTX	5.3^∗∗^	17 AMP	8.2^∗^	17CIP	3.7^∗^
60		16.8^∗^		4.0^∗^		3.4
30	21CTX	6.3	21 AMP	4.7	21CIP	2.4
60		2.4^∗^		3.5		3.8^∗^
30	22CTX	2.4	22 AMP	1.4	22CIP	1.8
60		2.7^∗^		1.8		1.7^∗^
30	23CTX	1.3	23 AMP	1.4	23CIP	1.2
60		1.3		1.3		2.0

*Donor represents the transconjugants named after the name or ESBL number of the original donor followed by the antibiotic used in the first conjugation experiment. The asterisks indicate statistical significance at level: ^∗^*P* ≤ 0.05, ^∗∗^*P* ≤ 0.01.*

## Discussion

The increasing occurrence of antibiotic resistance (AMR) among pathogenic bacteria is considered a major problem for public health in recent decades; and conjugation plays a major role in the spread of AMR (Zatyka and Thomas, 1998; Pfaffl, 2001; Sorensen et al., 2005; Bennett, 2008; Norman et al., 2009; Thomsen et al., 2016; von Wintersdorff et al., 2016). Even though many studies have focused on characterizing the impact of antibiotics on PT, the conclusions are not comparable (Barr et al., 1986; Bahl et al., 2004; Feld et al., 2008; Lopatkin et al., 2016). The possible reasons for these divergent results may be caused by different types and concentrations of antibiotics, different plasmids, and strains tested. A recent publication supports this hypothesis, as they also pointed out that antibiotics contribute to the increased HGT, but the level of HGT also relied on which donor and recipient cells were involved (Lu et al., 2017).

Extended spectrum beta-lactamases genes, and in particular CTX-M beta-lactamases, have become the most prevalent cephalosporin-resistance determinants in Enterobacteriaceae (Hawkey and Jones, 2009), and it was considered important to understand whether antimicrobial treatments may contribute to the spread of these genes. Therefore, 25 *E. coli* strains harboring different naturally occurring ESBL plasmids were selected to perform conjugation experiments. MG1655/pTF2 was included as we have previously shown that CTX could induce PT in this strain (Moller et al., 2017). Our main aim was therefore to investigate the effect of β-lactams on PT of the different ESBL plasmids. We chose to include CIP, as another antibiotic class to see whether we would get similar results, as CIP has previously been shown to increase PT (Shun-Mei et al., 2018). The strains encompassed all diverse ESBL genes and plasmids observed in ESBL positive *E. coli* from AMR surveillance in livestock in Denmark in 2015, and consequently showed uneven distribution of resistance genes, reflecting that *bla*_CTX–M–__1_ was the most common ESBL gene in *E. coli* from livestock (Meunier et al., 2006; Rodriguez et al., 2009). We found that antibiotic treatment significantly increased PT in eight ESBL-producing *E. coli*. In addition, at least four other *bla*_CTX–M–__1_-positive strains with IncI1 plasmid revealed an increased PT upon antibiotic exposure, however, not statistically significantly. We observed a high prevalence of antibiotic induced PT from strains with IncI1 plasmids, however, this was also the most common ESBL plasmid in our collection, present in 18 out of our 26 strains investigated. We observed no PT from 10 strains. Previous studies have shown that *traF* is essential in the formation of mating apparatus and conjugative transfer; and modification of any region of *traF* resulted in a loss of conjugative function (Waters et al., 1992; Lento et al., 2016; Huang et al., 2019). Analysis of the 10 strains from which no transconjugants was observed, showed that in 7 strains (ESBL 2, 7, 9, 13, 19, 20, and 25) the *traF* gene was absent. Additionally, ESBL19 lacked *traI*, *traL*, and *traM*. These deletions may be the cause of the lack of conjugation of these plasmids. Three of the plasmids (from ESBL 3, 15, and 24) encoded the *tra* genes and could potentially conjugate, but below detection limit. When examining the transconjugants, we investigated expression levels of five transfer genes involved in the conjugation process. *traF* and *traL* are involved in the pilus synthesis and assembly system, *traI* and *traM* participate in the DNA transfer system, and *pilS* is one of the pilus structural components. These genes were selected as they all play an important role in conjugation and have previously been shown to be up-regulated in bacteria exposed to CTX (Moller et al., 2017). We found these genes to be up-regulated in the transconjugants when exposed to antibiotics. It was furthermore demonstrated that this up-regulation of transfer associated genes correlated with an increase in PT frequency. Although the increased PT for some strains exposed to specific antibiotics did not increase beyond a statistically significant threshold, this non-significant increased PT still correlated to increased expression of the *tra* genes, supporting that antibiotics also induced PT in these strains (Table 3 and Figure 1). That we could show antibiotic increased PT when using the transconjugants as donors, indicated that antibiotic-increased PT was independent of the strains harboring the ESBL plasmids. When comparing the PT of the two rounds of conjugation, we observed that the frequency of PT of the second round of conjugation was higher than that of the first round of conjugation. This might be because the strains used as donors (*E. coli* J53-2 harboring plasmids) and recipients (*E. coli* J53-1) in the second round have high homology being resistant derivatives of the same K12 strain.

Previously, it had been shown that induction of the SOS-response by antibiotics enhanced transfer of plasmids and ICEs in *Vibrio cholerae*, *Aeromonas hydrophila*, and *S. aureus* (Barr et al., 1986; Beaber et al., 2004; Cantas et al., 2012). However, we have shown that CTX exposure increases transfer of a *bla*_CTX–M–__1_ encoding IncI1 plasmid in *E. coli* in a SOS-independent manner (Moller et al., 2017). We therefore also investigated the involvement of the SOS response, by comparing the expression of two genes associated with the SOS response, *recA* and *sfiA*, in the transconjugants with and without antibiotic exposure. We found that none of the typical SOS-response genes were induced by growth in the presence of CTX and AMP, but they were induced when the strains were exposed to CIP. This result supports previous findings showing that quinolones/CIP induce the SOS response (Piddock and Wise, 1987; Beaber et al., 2004). However, a recent study showed that even though CIP increased the expression of genes associated with the SOS response, there was no correlation with conjugation efficiency, and they concluded that sub-MIC CIP may promote conjugational PT by up-regulating the expression of conjugation associated genes via an SOS-independent mechanism (Shun-Mei et al., 2018). Since the strains are CTX and AMP resistant, but CIP sensitive, it could be suggested that only when exposed to CIP, the strains induce the SOS response to modulate their susceptibility. It is therefore currently not possible to conclude whether the CIP-induced PT and SOS-response occurs through dependent or independent regulatory pathways.

Previously published results (Moller et al., 2017) revealed that the resistance gene (*bla*_CTX–M–__1_) was required for CTX-induced *tra-*expression and PT. The mechanism behind the up-regulation remains elusive. A proteomic study of MG1655/pTF2 exposed to CTX only showed up-regulation of plasmid-encoded transfer-related proteins, the CTX-M-1 β-lactamase, and a hypothetical protein in response to the antibiotic. However, a proteomic approach may be to insensitive to determine the pathways involved, since various regulatory genes (e.g., two-component systems, regulatory RNAs, and regulatory proteins) may not show significant up-regulation, but yet be changed in expression. Further investigations on the regulatory mechanism(s) are therefore relevant.

## Conclusion

In conclusion, our findings reveal that exposure to different antibiotics can increase conjugative transfer of different naturally occurring β-lactam resistance plasmids in a strain-independent manner. Thus, antibiotic-induced conjugation transfer of ESBL plasmids appears to be a more common phenomenon in *E. coli*, at least for plasmids carrying the CTX-M *bla*-types. Since increased conjugation frequency will be an adverse effect of drug treatment of humans and animals, there is an urgent need to investigate the mechanisms underlying antibiotic-mediated increased conjugative PT, to be able to counteract the spread of resistance. Our findings have implications for evaluating the risks of antibiotic use, and for developing new antibiotic treatment protocols.

## Data Availibility

The raw data supporting the conclusions of this manuscript will be made available by the authors, without undue reservation, to any qualified researcher.

## Author Contributions

GL, LT, VB, and JO designed the study. GL and KB carried out the experiments. GL, VB, and LT analyzed the results. GL and LT wrote the manuscript. All authors commented on and approved the final version of the manuscript.

## Conflict of Interest Statement

The authors declare that the research was conducted in the absence of any commercial or financial relationships that could be construed as a potential conflict of interest.

## Footnotes

1
https://cge.cbs.dtu.dk/services/

2
https://pubmlst.org

3
http://pubmlst.org/plasmid/
