# Supplementary material for: Antibiotic-Induced, Increased Conjugative Transfer Is Common to Diverse Naturally Occurring ESBL Plasmids in Escherichia coli
Source: Front Microbiol. 2019 Sep 10;10:2119. doi: 10.3389/fmicb.2019.02119 (PMC6747055; doi:10.3389/fmicb.2019.02119)
Supplement: Supplementary file 1 [file Data_Sheet_1.docx]

Supplementary Material

**1 Supplementary Figures and Tables**

**1.1 supplementary Tables**

**Supplementary Table 1:** ENA (European Nucleotide Archive) accession information

| **Sample name** | **Study accession** | **Sample accession** | **Secondary sample accession** |
| --- | --- | --- | --- |
| ESBL 1 | PRJEB14641 | SAMEA4058360 | ERS1229470 |
| ESBL 2 | PRJEB14086 | SAMEA3993576 | ERR1417722 |
| ESBL 3 | PRJEB14086 | SAMEA3993616 | ERR1417762 |
| ESBL 4 | PRJEB14086 | SAMEA3993604 | ERR1417750 |
| ESBL 5 | PRJEB14641 | SAMEA4058366 | ERS1229476 |
| ESBL 6 | PRJEB14641 | SAMEA4058386 | ERS1229496 |
| ESBL 7 | PRJEB14086 | SAMEA3993618 | ERR1417764 |
| ESBL 8 | PRJEB14086 | SAMEA3993610 | ERR1417756 |
| ESBL 9 | PRJEB14086 | SAMEA3993613 | ERR1417759 |
| ESBL 10 | PRJEB14086 | SAMEA3993579 | ERR1417725 |
| ESBL 11 | PRJEB14086 | SAMEA3993585 | ERR1417731 |
| ESBL 12 | PRJEB14641 | SAMEA4058414 | ERS1229584 |
| ESBL 13 | PRJEB14641 | SAMEA4058431 | ERS1229541 |
| ESBL 14 | PRJEB14641 | SAMEA4058476 | ERS1229586 |
| ESBL 15 | PRJEB14641 | SAMEA4058418 | ERS1229528 |
| ESBL 16 | PRJEB14086 | SAMEA3993569 | ERR1417715 |
| ESBL 17 | PRJEB14086 | SAMEA3993566 | ERR1417712 |
| ESBL 18 | PRJEB14086 | SAMEA3993580 | ERR1417726 |
| ESBL 19 | PRJEB14086 | SAMEA3993593 | ERS1164703 |
| ESBL 20 | PRJEB14641 | SAMEA4058470 | ERS1229580 |
| ESBL 21 | PRJEB14641 | SAMEA4058384 | ERS1229494 |
| ESBL 22 | PRJEB14641 | SAMEA4058375 | ERS1229485 |
| ESBL 23 | PRJEB14641 | SAMEA4058391 | ERS1229501 |
| ESBL 24 | PRJEB14086 | SAMEA3993603 | ERR1417749 |
| ESBL 25 | PRJEB14641 | SAMEA4058426 | ERS1229536 |

**Supplementary table 2:** Minimal Inhibitory concentration **(**MIC) of ampicillin (AMP), cefotaxime (CTX) and ciprofloxacin (CIP) of donors and transconjugants from the first conjugation round.

| Strain ID | MIC-CTX  (mg/L) | MIC-AMP  (mg/L) | MIC-CIP  (mg/L) | Strain ID | MIC-CTX  (mg/L) | MIC-AMP  (mg/L) | MIC-CIP  (mg/L) |
| --- | --- | --- | --- | --- | --- | --- | --- |
| MG1655/pTF2 | 256 | 4000 | 0.008 | J53-2/pTF2 CTX | 32 | 4096 | 0.008 |
| ESBL 1 | 256 | 2400 | 0.008 | 4CTX | 256 | 3000 | 0.016 |
| ESBL 2 | 256 | 3000 | 0.016 | 4AMP | 512 | 2400 | 0.006 |
| ESBL 3 | 512 | 2400 | 0.006 | 4CIP | 512 | 2400 | 0.012 |
| ESBL 4 | 512 | 2400 | 0.012 | 10CTX | 256 | 3000 | 0.006 |
| ESBL 5 | 512 | 3400 | 0.006 | 10AMP | 64 | 2048 | 0.006 |
| ESBL 6 | 128 | 2400 | 0.006 | 10CIP | 256 | 3200 | 0.006 |
| ESBL 7 | 128 | 5000 | 0.012 | 12CTX | 128 | 4096 | 0.006 |
| ESBL 8 | 128 | 4000 | 0.012 | 12AMP | 256 | 4096 | 0.006 |
| ESBL 9 | 128 | 3400 | 0.012 | 12CIP | 256 | 4096 | 0.006 |
| ESBL 10 | 128 | 2400 | 0.008 | 17CTX | 256 | 2048 | 0.004 |
| ESBL 11 | 256 | 2048 | 0.006 | 17AMP | 128 | 2048 | 0.004 |
| ESBL 12 | 512 | 4000 | 1.024 | 17CIP | 256 | 2048 | 0.006 |
| ESBL 13 | 256 | 3800 | 0.008 | 21CTX | 128 | 2048 | 0.006 |
| ESBL 14 | 128 | 2048 | 0.006 | 21AMP | 256 | 2048 | 0.006 |
| ESBL 15 | 256 | 2048 | 0.006 | 21CIP | 16 | 2400 | 0.012 |
| ESBL 16 | 16 | 2400 | 0.012 | 22CTX | 512 | 3000 | 0.004 |
| ESBL 17 | 512 | 2048 | 0.008 | 22AMP | 512 | 3000 | 0.004 |
| ESBL 18 | 64 | 2000 | 0.003 | 22CIP | 512 | 3000 | 0.004 |
| ESBL 19 | 128 | 2000 | 0.004 | 23CTX | 128 | 2048 | 0.004 |
| ESBL 20 | 16 | 1024 | 0.008 | 23AMP | 128 | 2048 | 0.004 |
| ESBL 21 | 1024 | 5000 | 0.006 | 23CIP | 128 | 2048 | 0.004 |
| ESBL 22 | 256 | 2400 | 0.006 |  |  |  |  |
| ESBL 23 | 128 | 3000 | 0.006 |  |  |  |  |
| ESBL 24 | 64 | 2048 | 0.006 |  |  |  |  |
| ESBL 25 | 8 | 1024 | 0.256 |  |  |  |  |

Strain ID represents the donors and the transconjugants named after the name or ESBL number of the original donor followed by the antibiotic used in the first conjugation experiment.

**Supplementary Table 3.** Primer sequences for RT-qPCR.

| **Primer** | **Sequence** |
| --- | --- |
| nusG | for: 5’-GTCCGTTCGCAGACTTTAAC-3’ |
|  | rev: 5’-GCTTTCTCAACCTGACTGAAG-3’ |
| gapA | for: 5’-ACTGACTGGTATGGCGTTCC-3’ |
|  | rev: 5’-GTTGCAGCTTTTTCCAGACG-3’ |
| pilS | for: 5’-AGGGGCTGCTAAAAGGTAGC-3’ |
|  | rev: 5’-CTGTCATGGTCTTGGGTACG-3’ |
| traF | for: 5’-GACGTCGGAATTTCATTTGC -3’ |
|  | rev: 5’-TCCACACGCTGATATTTTGG -3’ |
| traI | for: 5’-CGGGAAAGCACACTTAATGC-3’ |
|  | rev: 5’-CTGGCGTGATATGAGCTACG-3’ |
| traL | for: 5’-ATGGTCACAACGTGAAAACG-3’ |
|  | rev: 5’-GAACTGGGGGAGGTTTATGC-3’ |
| traM | for: 5’-GGAGTCAGAATGATGCAATGG-3’ |
|  | rev: 5’-AGGGAGGAGATCTGTGAACG-3’ |
| recA | for: 5´-ACACCGGCGAGCAGGCACTGGAAA-3’ |
|  | rev:5´-ACGTGCCGCAAGGCCCATGTGA-3’ |
| sfiA | for: 5’- CGGGAATGGGTTCAGGCATCTGGGC-3’ |
|  | rev: 5’- TGCCCGTGCGTAAAGCGCGAACCA-3’ |
| dxs | for: 5’- CGAGAAACTGGCGATCCTTA-3’ |
|  | rev: 5’- CTTCATCAAGCGGTTTCACA-3’ |

**1.2 Supplementary Figures**

**
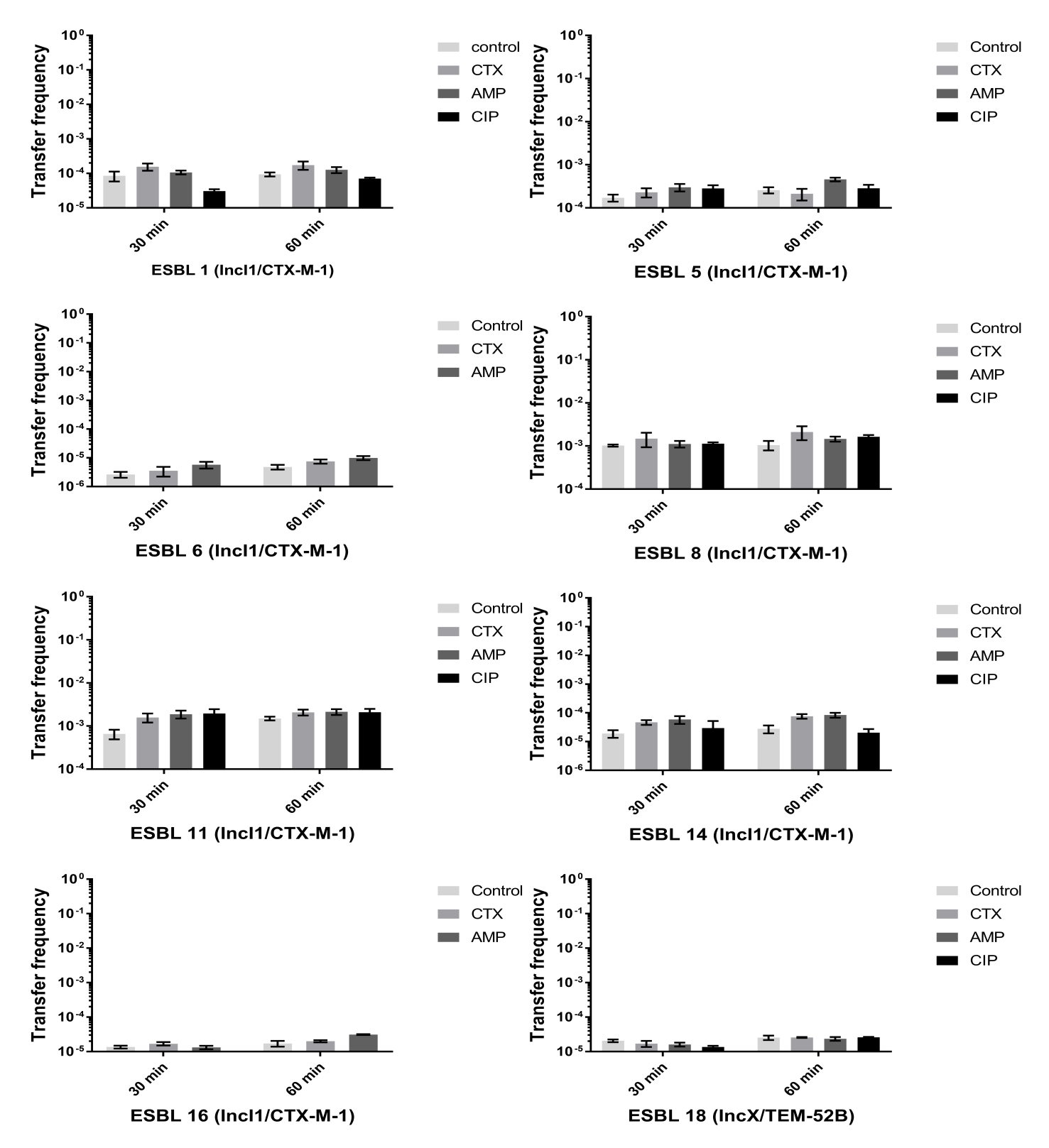
**

**Supplementary Figure 1**: Conjugation transfer frequency for different ESBL plasmids in different wild-type *E. coli* donor strains grown with or without (Control) antibiotics (CTX, cefotaxime; AMP, ampicillin; and CIP, ciprofloxacin) before the conjugation experiments. Names corresponds to donor name. 30 and 60 min refer to the time the donor and recipient had cell contact before the number of transconjugants was determined. No transconjugants were observed for ESBL 6 and ESBL 16 when pre-grown with CIP. The results shown are the means of three biological replicates with two technical replicates each and the error bars represent standard errors of the means.

**
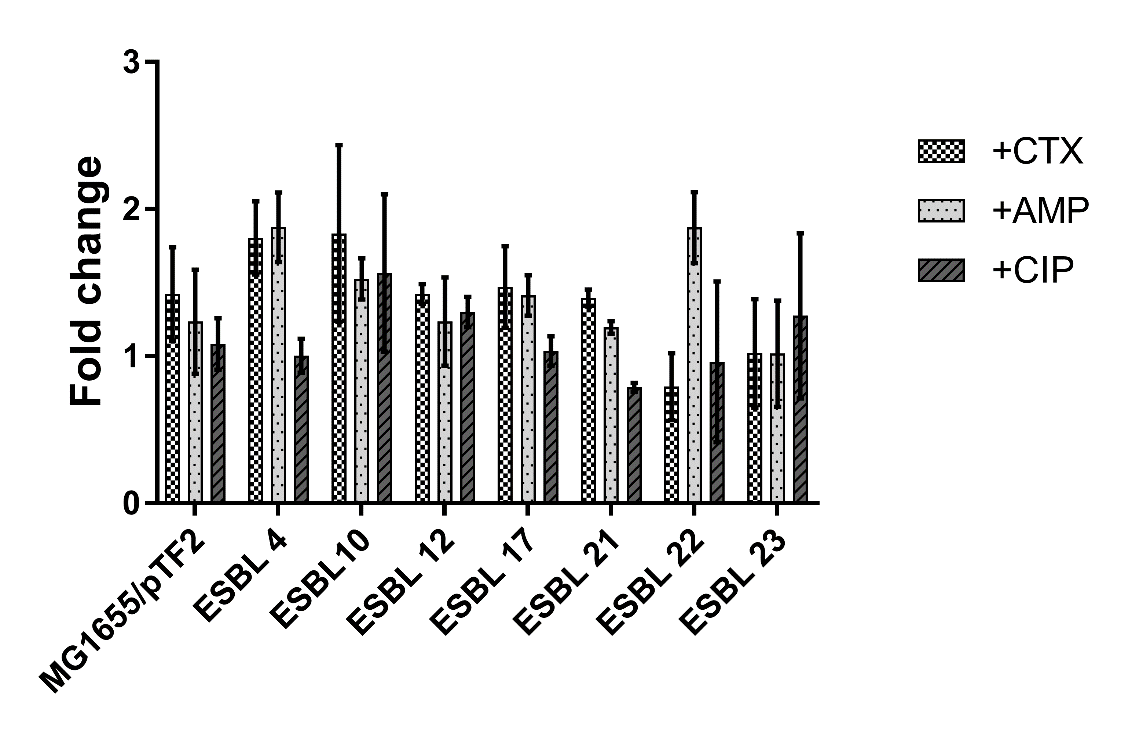
**

**Supplementary Figure 2**: Copy number analysis of different ESBL plasmids in different wild-type *E. coli* donor strains grown with antibiotics (CTX, cefotaxime; AMP, ampicillin; and CIP, ciprofloxacin). Data are presented as fold change relative to control without antibiotics. The results shown are the means of three biological replicates with two technical replicates each and the error bars represent standard errors of the means.
